# Ileal Atresia With Duplication Cyst Of Terminal Ileum: A Rare Association

**Published:** 2012-04-01

**Authors:** S Sinha, Yk Sarin, S Ramji

**Affiliations:** Department of Pediatric Surgery, Maulana Azad Medical College, New Delhi-110002.; 1 Department of Neonatology, Maulana Azad Medical College, New Delhi-110002.

**Keywords:** ileal atresia, duplication cyst

## Abstract

A case of ileal atresia in association with duplication of terminal ileum is being reported here.

## INTRODUCTION

Jejuno-ileal atresias and jejuno-ileal duplication cysts are uncommon gastrointestinal malformations. The association of these two malformations is extremely rare with very few reports in English literature [1-9]. We report a case of ileal atresia in association with ileal duplication cyst in a neonate. The relevant literature pertaining to the etiology of these lesions is discussed.

## CASE REPORT

A term female baby weighing 3130 grams was born by normal delivery to a second gravida mother in our hospital. Mother had a previous history of abortion at 2 months of gestation. At 36 weeks of gestation, antenatal sonogram revealed a massively dilated bowel loop; no other anomaly was detected. After birth, she developed features of low intestinal obstruction as evidenced by bilious nasogastric aspirates and air-fluid levels on plain X-ray abdomen. Post-natal ultrasonogram revealed a cyst in the right abdomen measuring 1.5 x 1.3 cm. Con-trast enhanced abdominal CT scan revealed grossly dilated bowel loops and a small retroperitoneal cyst of the same dimensions as above, adjacent to the right kidney (Fig.1). Contrast enema demonstrated a microcolon (Fig. 2). A preoperative diagnosis of intestinal atresia was made. The unusual location of the abdominal cyst ruled out the possibility of it being a cyst of ovarian, mesenteric or choledochal origin; the diagnosis that was considered at this stage was ‘incidental’ bowel duplication cyst that was unrelated to the bowel atresia.

**Figure:**
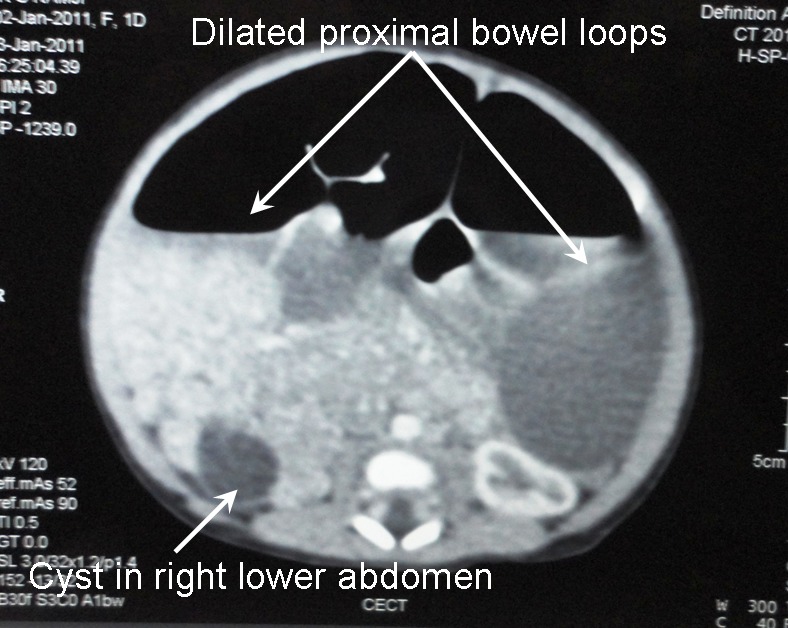
Figure 1: CT scan showing a cyst in the right lower abdomen with dilated proximal bowel loops.

**Figure:**
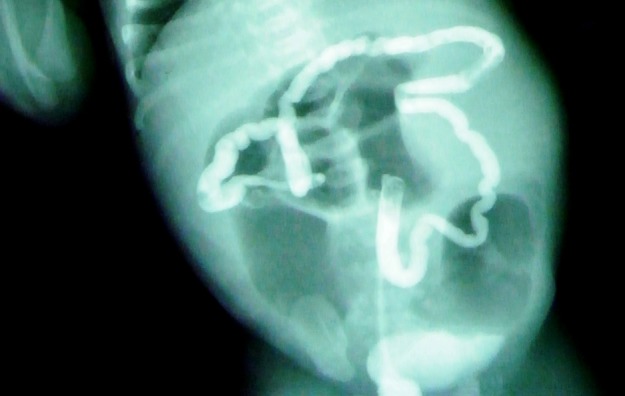
Figure 2: Contrast enema demonstrating microcolon.

The neonate was taken up for laparotomy at 28 hours of age and intestinal atresia was confirmed at surgery. However, the ileum distal to the atresia had formed a cocoon due to adhesions and was full of soft meconium pellets which were obstructing the free flow of saline into the caecum. There were tell-tale signs of a vascular event at the site of atresia with evidence of resorption of a bowel segment with a separate vessel supplying it. There was a non-communicating duplication cyst within the leaves of the bowel mesentery and sharing a common wall as well as blood supply with the terminal ileum, 7cm proximal to the ileocecal valve (Fig. 3). Since the cyst could not be separated from the bowel wall, two resection anastomoses were done at the site of excised duplication cyst and ileal atresia (after resecting about 15cm of dilated proximal bowel). The bowel distal to atresia was gently flushed with saline till all the meconium pellets were washed out prior to the anastomoses.

**Figure:**
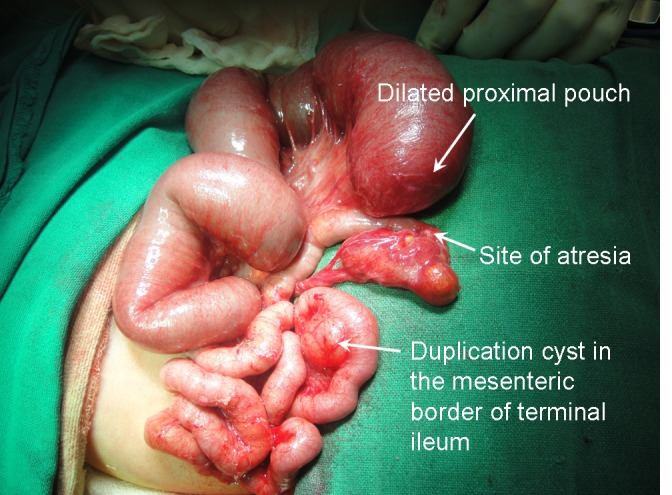
Figure 3: Intra-operative picture showing dilated proximal pouch, site of atresia and duplication cyst in mesenteric border of terminal ileum.

Postoperatively, the neonate was kept on total parenteral nutrition and continuous nasogastric suction for 10 days. Gradual enteral feeding was started thereafter and built up to full feeds. The baby was discharged on 15th postoperative day. The biopsy report was consistent with ileal duplication cyst. The cyst wall showed ulceration and chronic inflammation. The excised segment of atretic bowel showed marked bowel submucosal fibrosis and focal calcification. The excised intervening bowel segment was filled with necrotic and focally calcific material. The mucosa was replaced by granulation. Submucosa showed dense inflammation with organising exudate and foreign body giant cell reaction to hemosiderin. The surrounding muscularis showed focal thinning with serosal fibrosis. The child has been followed up for 1 year and is thriving well.

## DISCUSSION

The incidence of bowel duplication cysts in patients with jejuno-ileal atresias has been quoted as 0-4.7% [4,10]. Conversely, the incidence of jejuno-ileal atresias in series of jejuno-ileal duplications has been reported to be 10% [3].

Various eitiopathogenetic mechanisms have been proposed to explain the development of intestinal atresia and duplications and their unusual concurrence [1,2,7,11-14]. Mellish and Koop [1] suggested that environmental factors such as trauma or hypoxia in early fetal development were likely to be responsible when multiple duplications are found in association with anomalies such as malrotation or atresia. A mesenteric vascular accident has been implicated as a causative factor for both intestinal atresia and completely isolated duplication cyst [11,12]. This was supported by Favara et al. [2] who postulated that antenatal vascular accidents can result in four types of lesions depending on the severity of the accident and the subsequent healing: short bowel, intestinal stenosis, intestinal atresia, and enteric duplication. They also speculated that enteric duplication can occur when a segment of intestine is spared from necrosis and is nourished by the blood supply from the neighbouring inflamed intestine. Al Salem found evidence of ischemic insult in the form of intra¬abdominal calcification in a baby with multiple atresias and bowel duplication [13]. Similarly, Ratan et al suggested that differential healing of a segment of intestine affected by an antenatal vascular accident probably resulted in the simultaneous formation of intestinal atresia and a duplication cyst [7]. Sinha et al [6] suggested that atresias arise as a result of volvulus caused by the pre-existing duplication. As such intrauterine volvulus has been known to co-exist in as many 1/4th of all jejuno-ileal atresias [11]. Peterson et al described ileal intus-susception caused by an ileal duplication cyst, which may be one of the mechanisms of bowel duplica¬tion causing atretic changes in the adjacent bowel [14]. We believe that an antenatal vascular accident could be the aetiology of this association in our case as evidenced by presence of calcification.

Surgical management is straight forward. If the site of intestinal atresia is far away from the site of duplication, two separate resection-anastomoses are required as was done in this case.

## Footnotes

**Source of Support:** Nil

**Conflict of Interest:** None declared

